# Supplementary material for: In Vivo Emergence of Podovirus Resistance via tarS Mutation During Phage-Antibiotic Treatment of Experimental MSSA Endocarditis
Source: Viruses. 2025 Jul 25;17(8):1039. doi: 10.3390/v17081039 (PMC12390327; doi:10.3390/v17081039)
Supplement: Supplementary file 1 [file viruses-17-01039-s001.zip › Supplemental files.pdf]

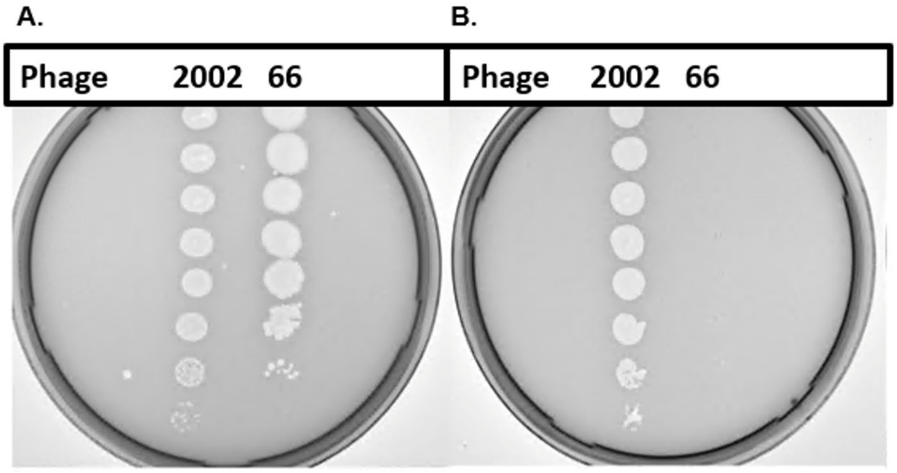

Figure S1. DDT patterns harbored by clones recovered form cardiac vegetations after 24 h treatment with the phage cocktail/flucloxacillin combination that were (**A.**) still susceptible to both phages of the cocktail and (**B.**) fully resistant to phage 66 but still susceptible to phage 2002.

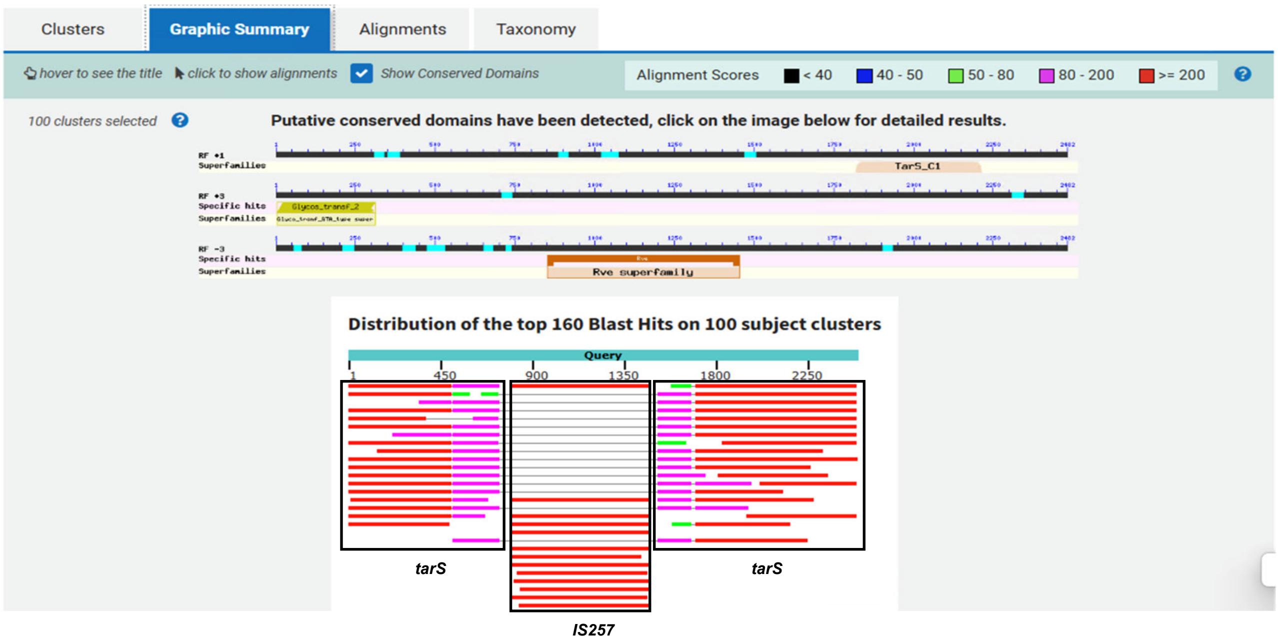

Figure S2. Schematic representation of the reconstituted genomic region of clone 16C02 encompassing the IS257 element inserted in *tarS* and graphic summary of the results obtained from blastx against the nr_clustered database of NCBI.

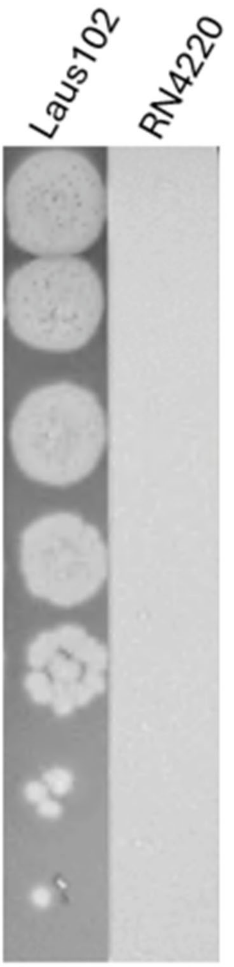

Figure S3. DDT of phage 66 on Laus102 and RN4220.

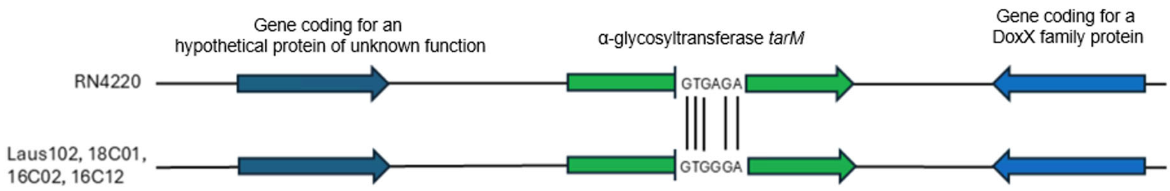

Figure S4. Schematic representation of *tarM* and its flanking genes carried by the genomes of the studied clones. Functions of the encoded proteins are indicated.
